# Nutritional Profiling, Phytochemical Composition and Antidiabetic Potential of *Taraxacum officinale*, an Underutilized Herb

**DOI:** 10.3390/molecules27175380

**Published:** 2022-08-24

**Authors:** Imtiyaz Murtaza, Omi Laila, Iqra Drabu, Ajaz Ahmad, Wafa Charifi, Simona M. Popescu, Sheikh Mansoor

**Affiliations:** 1 Biochemistry and Molecular Biotechnology Laboratory, Division of Basic Sciences and Humanities, SKUAST-K, J&K, Shalimar 190025, India; 2 Department of Clinical Pharmacy, College of Pharmacy, King Saud University, P.O. Box 2457, Riyadh 11451, Saudi Arabia; 3 Université de Paris, Institute Cochin, Inserm, CNRS, F-75014 Paris, France; 4 Department of Biology and Environmental Engineering, University of Craiova, 200585 Craiova, Romania; 5 Department of ACHG, Sher I Kashmir Institute of Medical Sciences, Soura, Srinagar 190011, India

**Keywords:** *T. officinale*, toxicity, phytochemicals, quercetin, antidiabetic, α-amylase, α-glucosidase

## Abstract

*Taraxacum officinale (T. officinale*), a wild vegetable with a number of health claims, has been mostly ignored and unexplored. The study aims to compare the nutritional, phytochemical as well as antidiabetic potential of fresh as well as shade-dried leaves of *T. officinale,* in order to recommend its best form as a dietary antidiabetic product. The results revealed that as compared to fresh leaves, the shade-dried leaves, in addition to possessing higher levels of carbohydrates, crude protein, crude fat, crude fiber, etc., also contain appreciable amounts of total phenols (5833.12 ± 4.222 mg/100), total flavonoids (188.84 ± 0.019 mg/100 g), ascorbic acid (34.70 ± 0.026 mg/100 g), β-carotene (3.88 ± 1.473 mg/100 g) and total chlorophyll (239.51 ± 0.015 mg/100 g) antioxidants. The study revealed the presence of medicinally important antidiabetic flavonoid quercetin present in *T. officinale* leaves. Among the three solvent systems used, the aqueous extract of shade-dried *T. officinale* leaves comparatively demonstrated potent antidiabetic activity under in vitro conditions in a dose-dependent manner via targeting α-amylase and α-glucosidase, the two potent enzymes of carbohydrate metabolism. Therefore, in addition to being a nutritious herb, the shade-dried leaves of *T. officinale* have great potential to suppress post-prandial glucose rise and can be better exploited through clinical trials to be used as a dietary intervention for better management of diabetes.

## 1. Introduction

Diabetes is one of the most common non-communicable diseases prevalent in humans, and a major cause of morbidity and premature mortality worldwide. According to the International Diabetes Federation (2019), diabetes is expected to increase by almost 51 percent by 2045 and is emerging as one of the serious public health challenges of modern times [1]. It leads to persistent hyperglycemia, insufficient insulin production or the resistance of cells to insulin action. Currently, the available conventional therapeutic options for diabetes management include the use of insulin or oral hyperglycemic drugs, which have many side effects and are accompanied by secondary failure rates [2]. Therefore, under such conditions, it becomes necessary to look for new therapeutic drugs or medicines that will be safer, cost effective and non-toxic. Traditionally, numerous edible plants with potential health-promoting activities are being recognized to have great potential for the management and prevention of diabetes due to the presence of various bioactive compounds [3,4]. The consumption of such bioactive rich foods can delay the complications and metabolic abnormalities associated with diabetes, especially by targeting the key enzymes of carbohydrate metabolism [5]. Thus, understanding their ethno-botanical information, chemical constituents and exploration of their probable mode of action may be quite helpful for the development of plant-based drugs for numerous health problems including diabetes. Nowadays, aggressive research is being carried out on such potent traditional edible herbs to be used as futuristic antidiabetic medicines, however, only after providing sufficient scientific evidence and justification for their therapeutic role.

The plant-biodiversity-rich union territory of Jammu and Kashmir of India is well known for traditionally using many edible vegetables as antidiabetic agents, which have great potential to act as novel therapeutics against diabetes, but only after being explored in scientific manner [6]. One of such potent underutilized traditional crops, *T. officinale*, has been considered to possess numerous medicinal properties, but is exploited at very small levels across the globe scientifically for its medicinal properties against various diseases like diabetes, cancer, inflammation, cardiovascular ailments, etc. [7,8]. In Kashmir, the herb is found growing naturally in almost all the hilly as well as plain areas. Therefore, the present research study examined the antidiabetic potential of fresh and shade-dried nutritionally rich *T. officinale* leaves as well as their possible putative antidiabetic mechanism using different solvent systems. The aqueous extract of shade-dried leaves possesses maximum antidiabetic potential via targeting the two potent enzymes of carbohydrate metabolism, i.e., α-amylase and α-glucosidase.

## 2. Results

### 2.1. Nutritional Composition

In the current study, the comparative nutritional analysis of fresh and shade-dried *T. officinale* leaves demonstrated variable results as presented in Table 1.

The results revealed that shade-dried leaf samples of *T. officinale* are nutritionally much more superior to fresh leaves. In the current study, the moisture content of fresh leaves was observed to be 81.94 ± 1.07% as compared to 7.04 ± 0.07% (91.4% decrease) present in shade-dried leaves. The ash content was found to be 12.28 ± 0.012% in shade-dried leaves as compared to 1.90 ± 0.06% present in fresh leaves and thus demonstrating a net increase of 546.3% after shade drying. The total carbohydrate content of fresh leaves was found to be 8.33 ± 0.678% as compared to 58.69 ± 0.015 in shade-dried leaves (604.5% increase). As compared to fresh leaves that contain 6.73 ± 0.052%, 2.49 ± 0.026% and 4.24 ± 0.047% total sugars, reducing sugars and non-reducing sugars, respectively, the shade-dried leaves contain 9.08 ± 0.035% (34.9% increase), 2.60 ± 0.017% (4.4% increase) and 6.50 ± 0.024% (53.3% increase) total sugars, reducing sugars and non-reducing sugars, respectively. Similarly, the crude protein content (3.82 ± 0.016%) and crude fat content (0.84 ± 0.017%) of fresh leaves was much lower as compared to 16.01 ± 0.025% (319.1% increase) and 4.29 ± 0.017% (410.7% increase), respectively, found in shade-dried leaves. Likewise, the shade-dried leaves contain 8.72 ± 0.023% crude fiber content as compared to 3.11 ± 0.012% present in fresh leaves, and thus demonstrate a net increase of 180.3% in crude fiber after shade drying.

### 2.2. Mineral Profiling

The results of the current study related to mineral profiling (in terms of calcium, iron, magnesium and potassium) of fresh as well as shade-dried *T. officinale* leaves are shown in Table 2.

The fresh leaves were found to possess 3.08 ± 0.021 mg/100 g iron, 392.76 ± 0.03 mg/100 g potassium, 192.06 ± 0.05 mg/100 g calcium and 37.93 ± 0.034 mg/100 g magnesium, respectively, as compared to 6.01 ± 0.28 mg/100 g iron, 405.75 ± 0.025 mg/100 g potassium, 204.68 ± 0.035 mg/100 g calcium and 45.76 ± 0.038 mg/100 g magnesium in shade-dried leaves. There was an almost 95.12%, 3.31%, 6.58% and 20.48% increase in iron, potassium, calcium and magnesium levels, respectively, in shade-dried leaves.

### 2.3. Phytochemicals

As shown in Table 3, in the current study, various bioactive constituents in the fresh as well as in shade-dried *T. officinale* leaves were analyzed.

A substantial amount of variation was observed to exist with respect to the concentration of various bioactive constituents (total phenols, total flavonoids, β-carotene, ascorbic acid, chlorophyll a, chlorophyll b, total chlorophyll) among the fresh and shade-dried *T. officinale* leaf samples. As is clear from Table 3, the total phenol content of fresh dandelion leaves was found to be 1707 ± 3.819 mg/100 g, which increased to 5833.12 ± 4.222 mg/100 g in shade-dried leaves. In parallel to this, the total flavonoid content of dried leaves increased to 188.84 ± 0.019 mg/100 g as compared to 179.44 ± 0.012 mg/100 g found in fresh leaves. The chlorophyll a, chlorophyll b and total chlorophyll content of fresh *T. officinale* leaves was observed to be 24.53 ± 0.027 mg/100 g, 20.69 ± 0.018 mg/100 g and 45.25 ± 0.015 mg/100 g, which increased to 178.03 ± 0.035 mg/100 g, 61.49 ± 0.049 mg/100 g and 239.51 ± 0.015 mg/100 g, respectively after shade drying. In contrast, the β-carotene (5.85 ± 0.167 mg/100 g) and ascorbic acid (39.95 ± 95 mg/100 g) content present in fresh *T. officinale* leaves decreased slightly to 3.88 ± 1.473 mg/100 g and 34.70 ± 0.026 mg/100 g, respectively in shade-dried leaves. However, the shade-drying method maximally retained the appreciable amount of these two photosensitive and heat labile important antioxidants in the leaves as compared to most of the other drying methods.

**Figure 1:**
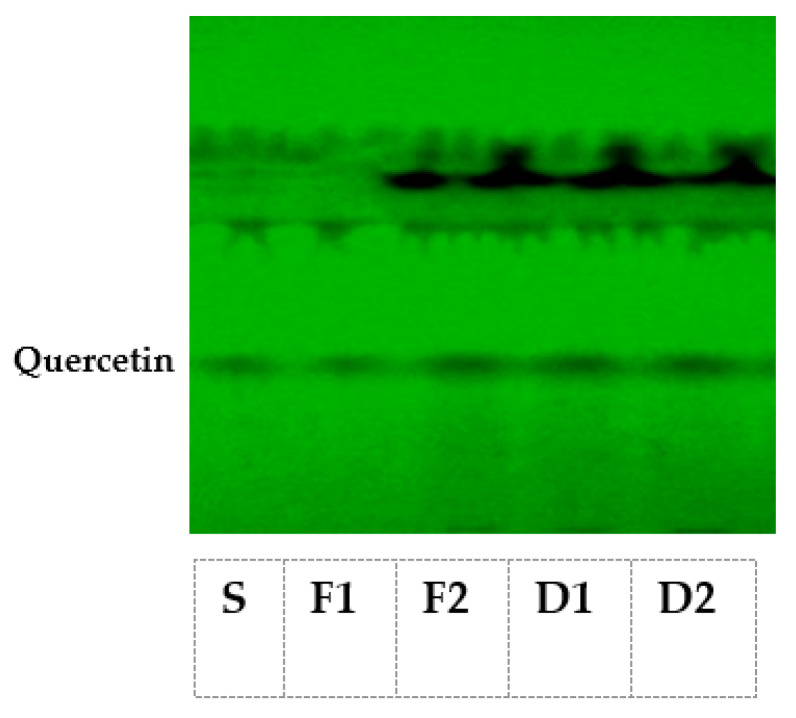
HPTLC chromatogram of *T. officinale* leaf extracts. S represents quercetin standard, F1 and F2 represent quercetin from fresh *T. officinale* leaf extracts, and D1 and D2 represent quercetin from shade-dried *T. officinale* leaf extracts.

As evident from Figure 1, two separate prominent bands for quercetin (corresponding to R_f value_ 0.57 ± 0.02) in fresh as well as dried *T. officinale* leaves each were observed, and authenticated by running the pure reference compound in parallel.

We could not observe any band for diosgenin in both fresh and shade-dried *T. officinale* leaf extracts, except the diosgenin in the reference compound (R_f value_ 0.69 ± 0.02).

### 2.4. Antidiabetic Potential of T. officinale Extracts

In the current investigation, in order to investigate the antidiabetic role of *T. officinale,* the two key carbohydrate digesting enzymes viz. α-amylase and α-glucosidase were targeted, while using the aqueous, ethanolic and methanolic extracts of both fresh and shade-dried *T. officinale* leaves as a source of inhibitors against these enzymes.

#### 2.4.1. α-Amylase Inhibition

**Figure 2:**
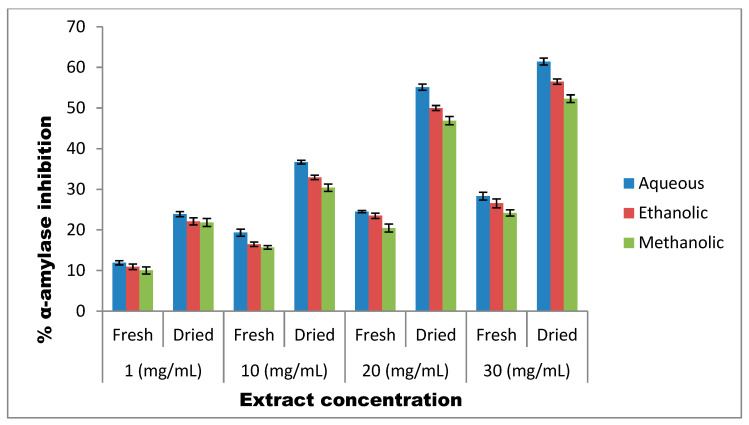
Inhibitory potential of *T. officinale* extracts against α-amylase.

Our results revealed that *T. officinale* leaves (fresh as well as shade-dried) proved to be potent antidiabetic agents and showed a moderate level of inhibitory activity against α-amylase. The dried extracts of *T. officinale* were found to be much more effective than the fresh *T. officinale* extracts. Among the different solvents tested, the aqueous extract was more powerful and showed the highest α-amylase inhibitory potential, followed by ethanolic and methanolic extracts. Further, the α-amylase inhibitory potential exhibited by all the three extracts was observed to be concentration-dependent as shown in Figure 2, and 30 mg/mL of each extract exhibited the highest inhibitory potential.

**Figure 3:**
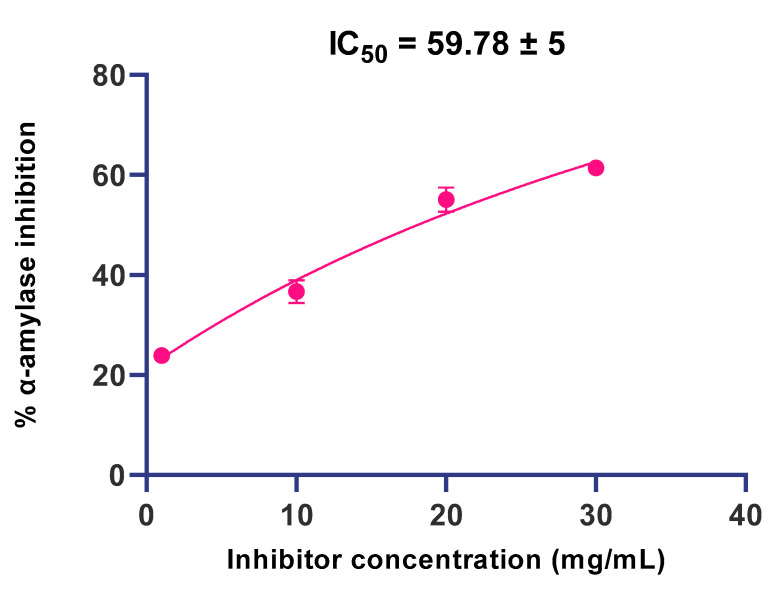
The IC_50_ value of aqueous extract of shade-dried *T. officinale* leaves against α-amylase.

Further, 1 mg/mL, 10 mg/mL, 20 mg/mL and 30 mg/mL of aqueous extracts of fresh leaves demonstrated 10.95%, 19.34%, 26.56% and 31.55% inhibition of α-amylase activity, respectively. Comparatively, the aqueous extract of shade-dried leaf samples proved more effective and caused 23.89% (118% increase), 36.68% (89.6% increase), 55.12% (107% increase) and 61.44% (94.7% increase) inhibition of α-amylase while using different concentrations i.e., 1 mg/mL, 10 mg/mL, 20 mg/mL and 30 mg/mL, respectively, of this extract. Likewise, the ethanolic extracts of fresh *T. officinale* leaves demonstrated 11.53%, 16.49%, 23.50% and 28.31% inhibitory potential against α-amylase as compared to 22.11%, 32.95%, 47% and 56.50% α-amylase inhibitory potential demonstrated by dried *T. officinale* leaves, at the respective concentrations of 1 mg/mL, 10 mg/mL, 20 mg/mL and 30 mg/mL. In the case of methanolic extracts of *T. officinale* leaves, the inhibitory potential of the fresh form against α-amylase activity was also found to be much lower, i.e., 10.95% 15.72%, 23.47% and 26.18% as compared to the dried form, which demonstrated 21.82%, 31.39%, 46.89% and 52.30% α-amylase inhibitory potential at the respective concentrations of 1 mg/mL, 10 mg/mL, 20 mg/mL and 30 mg/mL. Moreover, the IC_50_ of the aqueous extract of shade-dried *T. officinale* leaves against α-amylase was found to be 59.78 ± 5 (Figure 3).

#### 2.4.2. α-Glucosidase Inhibition

**Figure 4:**
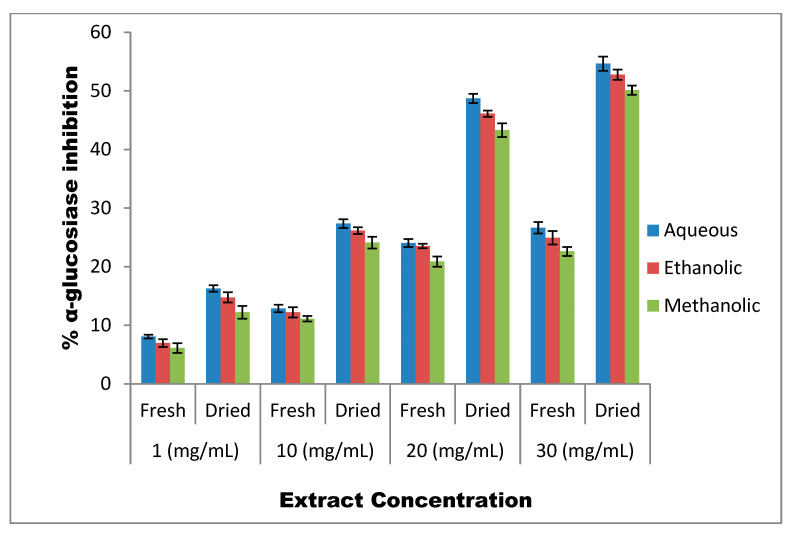
Percent α-glucosidase inhibitory potential of *T. officinale* extracts.

The study revealed that overall *T. officinale* leaves possess moderate α-glucosidase inhibitory potential, with shade-dried leaves exhibiting much higher α-glucosidase inhibitory potential than fresh *T. officinale* leaves (Figure 4). Among the different solvents tested, the aqueous extracts of *T. officinale* showed the highest inhibitory potential against α-glucosidase followed by ethanolic and methanolic extracts. The aqueous, ethanolic and methanolic extracts of fresh *T. officinale* leaves demonstrated variable inhibitory results, with 6.12%, 11.13%, 20.87% and 24.61% against α-glucosidase for aqueous extracts, at respective concentrations of 1 mg/mL, 10 mg/mL, 20 mg/mL and 30 mg/mL, followed by 8.07%, 12.88%, 24.04% and 26.65% for ethanolic extracts, and 6.97%, 12.22%, 23.52% and 24.95% for methanolic extracts, respectively. On the other hand, the shade-dried *T. officinale* aqueous, ethanolic and methanolic extracts demonstrated much higher inhibitory effects. The shade-dried aqueous extract demonstrated 12.22% (99.6% increase), 24.11% (116.6 increase), 43.31% (107.5% increase) and 50.14% (103.7% increase) inhibition against α-glucosidase, followed by 16.28%, 27.35%, 48.72% and 54.65% for ethanolic extracts and 14.76%, 26.16%, 46.12% and 52.77% for methanolic extracts, at respective concentrations of 1 mg/mL, 10 mg/mL, 20 mg/mL and 30 mg/mL, respectively.

**Figure 5:**
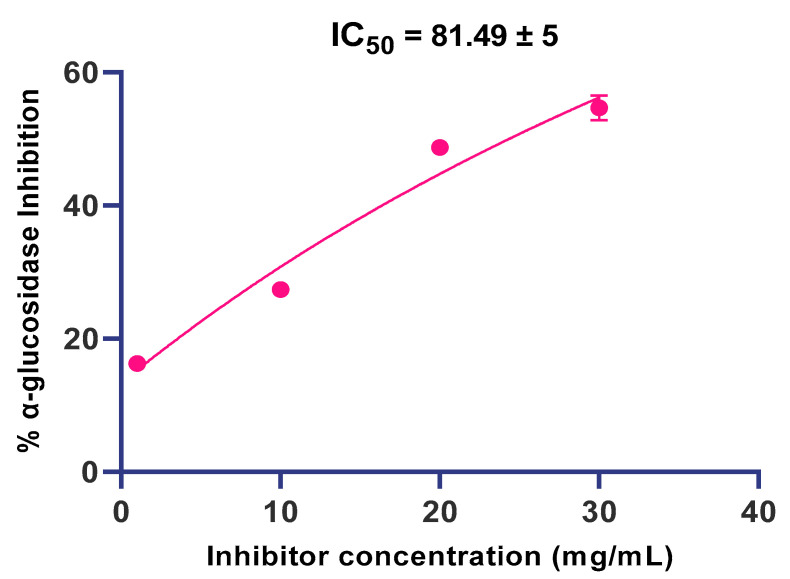
The IC_50_ value of aqueous extract of shade-dried *T. officinale* leaves against α-glucosidase.

Further, as shown in Figure 4, the inhibitory potential of all the three extracts (ethanolic, methanolic and aqueous) of fresh as well as dried dandelion leaves was found to be concentration-dependent and the highest inhibition against α-glucosidase was demonstrated by 30 mg/mL. The IC_50_ of aqueous extracts of shade-dried *T. officinale* leaves against α-glucosidase was found to be 81.49 ± 5 (Figure 5).

### 2.5. Correlation Analysis

As shown in Table 4 and Table 5, a strong positive correlation was established between the α-amylase inhibitory activity and the total phenols, total flavonoids, β-carotene and ascorbic acid, present in fresh as well as in dried *T. officinale* leaves.

Likewise, a strong positive correlation was established between the α-glucosidase inhibitory activity and the total phenols, total flavonoids, β-carotene and ascorbic acid, present in fresh as well as in dried *T. officinale* leaves (Table 4 and Table 5). The correlation study has been performed as per the student *t*-test (*t*-test) using the IBM SPSS Statistics for Windows, version 23.0 (IBM Corp., Armonk, NY, USA)

## 3. Discussion

Wild edible food plants have served since ancient times either as an ingredient of normal diets or as an alternative during situations of food scarcity [9]. These wild plants have been reported to be a rich source of nutrients (including vitamins, minerals and trace elements) and secondary metabolites, with potential health benefits. Such types of plants act as an important source of therapeutic material to maintain human health and have been used to prevent and treat various human diseases since ancient times [10]. One such medicinal herb, *T. officinale* (commonly known as dandelion), has nowadays gained a great deal of attention worldwide, due to its marked nutritional and medicinal potential [7,11]. The literature suggests *T. officinale* has been considered to be an aperient, stimulant, stomachic, tonic, antidiabetic and detoxicant in the traditional system of medicines [8,12]. However, *T. officinale* has remained highly unexplored scientifically for such health claims. Therefore, the current investigation was carried out to evaluate the nutritional, phytochemical and antidiabetic potential of fresh as well as shade-dried leaves of *T. officinale,* in order to use its best form as a dietary intervention for the management of diabetes. In the present era, more attention is being paid to exploit the possibilities of traditional/underutilized plant resources with possible health claims, and promote their utilization as alternate nutritive food crops as well as alternative medicines [13,14]. The results of the current study revealed that there exists a great deal of variation in the nutrient levels among the fresh and shade-dried *T. officinale* leaf samples, with the dried form to be nutritionally much more superior (Table 1) and the results are consistent with previous reports [15,16,17,18,19,20]. Research studies have also shown that mineral deficiencies are emerging rapidly across the world, especially in developing countries [21]. The high nutritional and mineral content of the shade-dried form of *T. officinale* leaves observed in this study suggests that this ignored crop should be included in the daily foods of poor sections of the population who cannot afford costly cultivated vegetables, in order to address the malnutrition and hidden hunger deficiency problems in developing countries such as India.

The vast and versatile pharmacological effects of medicinal plants are basically dependent on the significant levels of some specific phytochemical constituents or secondary metabolites present in them [22]. These bioactive compounds (in terms of phenolic acids, flavonoids, steroids, terpenoids, etc.) have been reported to act either individually or synergistically in alleviating several ailments (from migraines to cancer) in the traditional system of medicines or folklore uses, and provide a source of various lead compounds for the production of different medications for the treatment of various diseases in modern times [23]. Our results indicate a substantial amount of variation in the concentration of various bioactive constituents (in terms of total phenols, total flavonoids, β-carotene, ascorbic acid, chlorophyll a, chlorophyll b and total chlorophyll) among the fresh and shade-dried *T. officinale* leaf samples. As far as phenolic compounds are concerned, they are ubiquitously present in plant foods and their high dietary intake has been linked with various health benefits including the decrease in the incidence of various chronic diseases including diabetes [24,25]. Thus, the exploitation of phenolic rich foods will be quite health beneficial. As clear from Table 3, the total phenol content of fresh *T. officinale* leaves increased drastically after shade drying, with a net increase of almost 241.7% on drying. Among phenolics, flavonoids represent a large family of polyphenolic compounds and their dietary intake has been linked with a slew of potential health benefits in humans [26]. The total flavonoid content of shade-dried *T. officinale* leaves also increased to almost 5.2% as compared to fresh leaves. Naturally occurring plant pigments or phytochemicals including chlorophyll, carotenoids and vitamin C exhibit numerous pharmaceutical and therapeutic effects, including antioxidant, antibacterial, antidiabetic and anticancer activities [27]. Several studies have reported the direct relationship between natural pigments and their bioactivities. Chlorophyll pigment by itself does not contain any nutrients aside from magnesium; however, liquid extracts of chlorophyll have very healthy nutritional profiles. Chlorophyll is an antioxidant and has been reported to act as a hypoglycemic agent due to the inhibition of free radicals [28]. After shade drying, the total chlorophyll content of *T. officinale* leaves increased almost 429.3% and this form can thus act as a better antihyperglycemic agent. On the other hand, after shade drying, the β-carotene and ascorbic acid content of shade-dried *T. officinale* leaves decreased and indicated an almost 33.6% decrease in β-carotene content and a 13.1% decrease in ascorbic acid content. These results are in strong agreement with the previous findings, showing an overall increase in the majority of these phytoconstituents after shade drying and a slight decrease in some of the thermolabile compounds such as β-carotene and ascorbic acid [20,27,28,29,30,31,32]. The overall results demonstrate shade-dried *T. officinale* leaves to be the best source of phytochemicals and can be better exploited as a health-promoting agent against various oxidative stress-related diseases including diabetes.

There are reports that *T. officinale* leaves are rich in saponins and flavonoids, therefore, in this study, HPTLC analysis for the detection of bioactive steroidal saponin in the form of diosgenin and flavonoid as quercetin were analyzed, as both of these two phytochemicals have been reported to be effective against a variety of pathologies including diabetes [33]. In this study, fresh and dried *T. officinale* leaves after subjecting to HPTLC demonstrated the presence of only quercetin (corresponding to R_f value_ 0.57 ± 0.02) in both fresh as well as shade-dried leaves. These results are in strong agreement with the previous reports indicating the presence of quercetin in other *Taraxacum* species [34]. The results clearly indicated that diosgenin is most probably absent in the dandelion leaves. The study thus clearly indicates that due to presence of quercetin, *T. officinale* can act as potent hypoglycemic agent [35].

The inhibition of α-amylase and α-glucosidase, the two key enzymes of carbohydrate metabolism, is nowadays considered as a novel strategy for the modulation of postprandial hyperglycemia associated with diabetes [36]. At present, oral hypoglycemic drugs, such as acarbose, voglibose, miglitol, metformin, etc., constitute the predominant line of therapy that mostly act as α-amylase and α-glucosidase enzyme inhibitors of carbohydrate metabolism. Unfortunately, these allopathic drugs are associated with a number of mild side effects such as diarrhea, bloating, flatulence, cramping, abdominal pain and some severe health related issues [37]. Therefore, natural α-amylase, α-glucosidase and invertase inhibitors from plants are gaining much attention and offer an effective therapy for the treatment and management of postprandial hyperglycemia linked to diabetes [38,39]. The main aim of this research investigation is to explore the different extracts of fresh as well as shade-dried *T. officinale* leaves prepared in different solvent systems for their antidiabetic potential (in terms of α-amylase and α-glucosidase inhibition) so as to recommend the best form of this nutritionally rich wild herb as an antidiabetic food in a routine diet or as an alternative source of antidiabetic agent(s)/drug(s) for future use. Therefore, in the current investigation, α-amylase and α-glucosidase, the two key carbohydrate digesting enzymes, were targeted, while using the aqueous, ethanolic and methanolic extracts of fresh and shade-dried *T. officinale* extracts as inhibitors of these enzymes.

As far as α-amylase is concerned, our results revealed that *T. officinale* leaves (fresh as well as dried) possess moderate levels of inhibitory activity against this enzyme. The dried extracts of *T. officinale* were found to be much more effective than fresh *T. officinale* extracts. Among the different extracts tested, the aqueous extract was more powerful and showed the highest α-amylase inhibitory potential, followed by the ethanolic and methanolic extracts. Crude plant extract is a mixture of a multitude of bioactive constituents with diverse chemistries and pharmacological activities at different concentrations. If a constituent is effective at a low concentration, a gradual increase in its concentration may gradually reduce its efficacy. However, the potency or efficiency of the selected crude extract is still questionable at a low dosage and can work best at higher concentrations. At much higher concentrations, an active substance could blunt the activity of a second bioactive constituent. Furthermore, in some cases, the activity of a bioactive compound manifested at a certain concentration could be modified at a different concentration, owing to the overriding of the original effect elicited. Therefore, in the current investigation, the different concentrations of *T. officinale* extract prepared in three different solvents were included, in order to determine the optimal concentration of the selected extract in a particular solvent. Our results showed that the α-amylase inhibitory potential exhibited by all the three extracts was concentration-dependent and 30 mg/mL shade-dried *T. officinale* exhibited the highest α-amylase inhibitory potential, with 94.7% increased potential than the fresh one. These results corroborate well with the observations of Odhav et al. [40] who reported almost 71.95% α-amylase inhibition (at 5 mg/mL concentration) by aqueous *T. officinale* extract. The α-amylase inhibitory action of these extracts may be basically attributed to the hydrophilic character of the solvent tested and the structural affinity of α-amylase enzyme towards hydrophilic ligands/substrates [41].

As far as α-glucosidase is concerned, our results revealed that *T. officinale* leaves also possess moderate α-glucosidase inhibitory potential, and the dried form exhibited higher α-glucosidase inhibitory potential than the fresh *T. officinale* leaves. Among the different solvents tested, the aqueous *T. officinale* extract showed the highest inhibitory potential against α-glucosidase followed by the ethanolic and methanolic extracts. Further, the inhibitory potential of all the three extracts (ethanolic, methanolic and aqueous) of fresh as well as shade-dried leaves was found to be concentration-dependent, and the highest inhibition of 50.14% against α-glucosidase was demonstrated by the 30 mg/mL shade-dried aqueous leaf extract (103.7% increase) as compared to only 24.6% inhibition by the 30 mg/mL fresh aqueous leaf extract. These results are in agreement with the findings of Vadivel and Biesalski, who reported almost 65.13% α-glucosidase inhibition by methanolic *Acacia nilotica* extract [42]. Further, aqueous *Salacia-oblonga* extract was also reported to cause almost 68.5% α-glucoside inhibition at 100 mg/mL concentration [43]. The higher inhibitory property of shade-dried *T. officinale* aqueous leaf extracts against α amylase and α-glucoside may be linked to the presence of potent phytochemicals evaluated in this study as indicated in the correlation matrix tables.

## 4. Materials and Methods

### 4.1. Collection of Plant Material

The leaf samples of *T. officinale* at their edible growth stage were collected from the Division of Vegetable Science, SKUAST-K, Kashmir, J&K, India. The collected samples were surface sterilized first with sodium hypochlorite, followed by thorough rinsing with distilled water. A half portion of the collected samples was shade-dried, and both fresh as well as dried samples were subjected to further investigations.

### 4.2. Nutritional Analysis

We were interested in retaining the maximum content of thermolabile and photosensitive constituents possessing antioxidant and antidiabetic activity, such as β-carotene, ascorbic acid, etc., in *Taraxacum officinale* leaves after drying, so that in this form it can be used to develop a product with maximum retainment of bioactive constituents and bioactivities. The nutritional composition of shade-dried as well as fresh leaf samples of *T. officinale* was analyzed while using standard methods of AOAC [44]. The ash content of samples was determined by heating them at 500 °C in a muffle furnace for about 5–6 h, and total moisture content was determined by drying the fresh sample in the oven at 100–110 °C until it attained constant weight. The crude fat content of dried leaf samples was estimated by using a Soxhlet apparatus and petroleum ether (60–80 °C) as an extraction solvent. The crude protein content was determined by the micro-Kjeldahl method. The estimations of crude fiber content, total soluble sugars, reducing sugars, non-reducing sugars and total carbohydrates were determined by methods reported in Thimmaiah [45]. Crude fat refers to the crude mixture of fat-soluble material present in the sample and is commonly extracted in anhydrous ether while using the dry Soxhlet extraction method. Crude fiber does not mean dietary fiber, but refers to the residue (primarily cellulose and lignin) remaining after food is treated with acid and alkali.

### 4.3. Mineral Profiling

For mineral profiling, 1 g of *T. officinale* leaf samples was mixed separately with 20 mL of di-acid mixture of nitric acid and perchloric acid (9:4). The mixture was kept undisturbed for an overnight period and the digestion process was conducted at 115–118 °C until a watery transparent liquid was obtained. The liquid was filtered and the final volume was made up to 50 mL using double distilled water. Each sample was finally subjected to Atomic Absorption Spectroscopy (AA 800, Perkin-Elmer, Rodgau, Germany) for the estimation of Calcium (Ca), potassium (K), magnesium (Mg) and iron (Fe).

### 4.4. Phytochemical Analysis

#### 4.4.1. Total Phenols

The total phenolic content of leaf samples was evaluated by using Malick and Singh’s method [46]. The sample extract (0.1 mL) was combined with double distilled water (2.9 mL) followed by the addition of the Folin-Ciocalteu reagent (0.5 mL). The samples were incubated for 3 min and immediately 2 mL of sodium carbonate (20%) was added. The samples were vortexed and kept in a water bath (100 °C) for 1 min. Finally, the absorbance of samples was measured (650 nm) against the reagent blank, and total phenolic content was determined by using the catechol calibration curve.

#### 4.4.2. Total Flavonoids

The total flavonoid content of leaf samples was determined by the method reported by Lallianrawna et al. [47]. Briefly, 500 µL of leaf extract was combined with 75 µL of 5% sodium nitrite (NaNO_2_) and incubated for 6 min, followed by the addition of 150 µL of 10% aluminum chloride and incubated again for a further 6 min at room temperature. The reaction was finally terminated by adding 500 µL of 1M NaOH and the total volume was made up to 2.5 mL with deionized water in each tube. The absorbance of each sample was measured at 510 nm against reagent blank, and total flavonoids were determined by using the quercetin calibration curve.

#### 4.4.3. Ascorbic Acid

The ascorbic acid content of fresh as well as shade-dried leaf samples was determined by the volumetric method of Sadasivam and Theymoli [48]. The indicator dye 2,6-dichlorophenol-indophenol was used for the purpose, and the results were expressed on both fresh and dry weight basis.

#### 4.4.4. β-Carotene

The β-carotene content in the leaf samples was determined by Nagata and Yamashita’s method [49]. Briefly, 10 g of sample was homogenized in 100 mL of methanol, filtered and finally dried in a rotatory evaporator at 45 °C. Finally, 10 mL of acetone-hexane mixture (4:6) was added to the 100 mg of dried extract and after vigorous shaking for 1 min, the mixture was filtered again. The absorbance of the filtrate was measured at 453 nm, 505 nm, 645 nm and 663 nm. The β-carotene content in each sample was finally calculated by using the following equation:

β-carotene (mg/100 mL) = 0.216 (A_663_) − 0.304 (A_505_) + 0.452 (A_453_)

#### 4.4.5. Total Chlorophyll Content

The chlorophyll content of fresh as well as shade-dried *T. officinale* leaf samples was determined by Sadasivam and Manickam’s method [50], while using 80% acetone as an extraction solvent. The chlorophyll content of each sample (mg chlorophyll per g tissue) was estimated by applying the following formulae:

mg Chlorophyll a/g tissue = 12.7(A663) − 2.69(A645) × V/1000 × W

mg Chlorophyll b/g tissue = 22.9(A645) − 4.68(A663) × V/1000 × W

mg total Chlorophyll = 20.2(A645) + 8.02(A663) × V/1000 × w

where A = absorbance at specific wavelengths, V = final volume of chlorophyll extract in 80% acetone and W = fresh or dry weight of the tissue extracted.

#### 4.4.6. Diosgenin and Quercetin

The diosgenin and quercetin content of *T. officinale* samples was analyzed by using the method described by Laila et al. [51]. In 50 mL of acidified absolute ethanol, 1 g of leaf sample was refluxed at 100 °C for 60 min. The solution was cooled down, filtered and finally concentrated in a rotatory evaporator at 50 °C. To obtain concentrate, 25% ammonia solution (pH > 12) was added to make it alkaline and the resultant solution was diluted with double volume of distilled water. The mixture was thrice re-extracted with dichloromethane (DCM). The DCM extract was then sequentially washed first with 2 mL of 0.1 M sodium hydroxide and finally with 1 mL of distilled water. The extract obtained was evaporated to dryness at 50 °C using a rotary evaporator followed by dissolving the dried sample in 5 mL methanol. The reconstituted solution was filtered through a polypropylene membrane filter (0.45 μm) and subjected directly to HPTLC analysis.

### 4.5. In Vitro Antidiabetic Activity

#### 4.5.1. Preparation of Extract

Fresh as well as shade-dried *T. officinale* leaves (10 g each) were extracted by the maceration method, using a sufficient quantity of selected solvents (i.e., distilled water, ethanol and methanol). The resultant extracts were filtered and concentrated using a vacuum evaporator (40 °C). The dried extracts were transferred to a glass container followed by blowing the nitrogen in the container, and finally the container was weighed to calculate the yield of each extract obtained. The extracts obtained were redissolved in their respective solvents (distilled water, ethanol and methanol) to prepare the different concentrations (1 mg/mL, 10 mg/mL, 20 mg/mL and 30 mg/mL) of each extract of *T. officinale* and access their in vitro antidiabetic activities, in terms of the inhibition of α-amylase and α-glucoside activities.

#### 4.5.2. α-Amylase Inhibition Assay

The in vitro-based α-amylase inhibitory activity of *T. officinale* extract was determined by using the method developed by Worthington et al. [52]. The IC_50_ values of *T. officinale* extracts against α-amylase activity were calculated by using GraphPad Prism version 8 (GraphPad Software, Inc., La Jolla, CA, USA) statistical software. IC_50_ is defined as the concentration of *T. officinale* leaf extract required to inhibit 50% of α-amylase enzyme activity.

#### 4.5.3. α-Glucosidase Inhibition Assay

The in vitro based α-glucosidase inhibitory activity of *T. officinale* extract was determined by using the method developed by Worthington [53]. The IC_50_ values of *T. officinale* extracts against α-glucosidase activity was calculated by using GraphPad Prism version 8 (GraphPad Software, Inc., La Jolla, CA, USA) statistical software. IC_50_ is defined as the concentration of *T. officinale* leaf extract required to inhibit 50% of α-glucoidase enzyme activity.

### 4.6. Statistical Analysis

The data generated in this study were analyzed by using the IBM SPSS Statistics for Windows, version 23.0 (IBM Corp., Armonk, NY, USA) and applying chi-square Student’s *t-*test (*t-*test) and one-way analysis of variance (ANOVA).

## 5. Conclusions

In conclusion, the results of the current study clearly demonstrated that the aqueous extract of shade-dried *Taraxacum officinale* leaves is not only a good source of various vital nutrients, but can also act as a potential antidiabetic food-based medicine by targeting α-amylase and α-glucosidase, the key enzymes of carbohydrate metabolism, via bioactive compounds that seem to be involved in the inhibition of these enzymes. However, further research is needed before phytotherapy for diabetes through the aqueous extract of shade-dried *Taraxacum officinale* leaves can be advanced to the clinic.

## Figures and Tables

**Table 1:** Comparative analysis of proximate composition of *T. officinale* leaves (fresh and shade-dried).

Parameter	Fresh	Shade-Dried	T_cal_
Moisture (%)	81.94 ± 1.07	7.04 ± 0.07	103.05 **
Ash (%)	1.90 ± 0.006	12.28 ± 0.012	778.75 **
Carbohydrates (%)	8.33 ± 0.678	58.69 ± 0.015	74.26 **
Crude protein (%)	3.82 ± 0.016	16.01 ± 0.025	593.24 **
Crude fat (%)	0.84 ± 0.017	4.29 ± 0.017	202.78 **
Crude fiber (%)	3.11 ± 0.012	8.72 ± 0.023	297.33 **
Total sugars (%)	6.73 ± 0.052	9.08 ± 0.035	66.41 **
Reducing sugars (%)	2.49 ± 0.026	2.60 ± 0.017	5.15 **
Non-reducing sugars (%)	4.24 ± 0.047	6.50 ± 0.024	132.97 **

Results are expressed as mean ± SD and are the average of triplicates. ** indicates significance level at *p* < 0.01 and T_cal_ is the *t*-statistic for the data obtained.

**Table 2:** Comparative analysis of mineral content of *T. officinale* leaves (mg/100 g).

Parameter	Fresh	Dried	T_cal_
Iron	3.08 ± 0.021	6.01 ± 0.28	117.59 **
Potassium	392.76 ± 0.03	405.75 ± 0.025	607 **
Calcium	192.06 ± 0.05	204.68 ± 0.035	302.07 **
Magnesium	37.93 ± 0.034	45.76 ± 0.038	211.02 **

Results are expressed as mean ± SD and are the average of triplicates. ** indicates significance level of *p* < 0.01. T_cal_ is the *t*-statistic for the data obtained.

**Table 3:** Comparative analysis of bioactive constituents of *T. officinale* leaves.

Parameter	Fresh	Dried	T_cal_
Total phenols (mg/100 g)	1707 ± 3.819	5833.12 ± 4.222	724.68 **
Total flavonoids (mg/100 g)	179.44 ± 0.012	188.84 ± 0.019	424.83 **
β-carotene (mg/100 g)	5.85 ± 0.167	3.88 ± 1.473	3.0602
Ascorbic acid (mg/100 g)	39.95 ± 0.052	34.70 ± 0.026	141.34 **
Chlorophyll-a (mg/100 g)	24.53 ± 0.027	178.03 ± 0.035	3441.93 **
Chlorophyll-b (mg/100 g)	20.69 ± 0.018	61.49 ± 0.049	676.87 **
Total chlorophyll (mg/100 g)	45.25 ± 0.015	239.51 ± 0.015	8992.49 **

Results are expressed as mean ± SD and are the average of triplicates. ** indicates a significance level of *p* < 0.01. T_cal_ is the *t*-statistic for the data obtained.

**Table 4:** Correlation matrix between different variables and bioactive constituents (fresh *T. officinale* leaves).

Variables	Total Phenols	Total Flavonoids	β-Carotene	Ascorbic Acid
α-amylase inhibitory potential	0.96	0.94	0.90	0.91
α-glucosidase inhibitory potential	0.97	0.93	0.89	0.92

**Table 5:** Correlation matrix between different variables and bioactive constituents (dried *T. officinale* leaves).

Variables	Total Phenols	Total Flavonoids	β-Carotene	Ascorbic Acid
α-amylase inhibitory potential	0.98	0.96	0.87	0.88
α-glucosidase inhibitory potential	0.99	0.95	0.86	0.89

## Data Availability

Not applicable.

## Sample Availability

Samples of the compounds are available from the authors.
